# Effectiveness of combination therapy with nifedipine GITS: a prospective, 12-week observational study (AdADOSE)

**DOI:** 10.1186/s12872-015-0037-x

**Published:** 2015-05-09

**Authors:** Ahmed K. Motaweih, Elena Usova, Wajid Hussain, Ziad Dello, Birgit Schmidt, Thomas Petri

**Affiliations:** Cardiovascular Medicine Department, Faculty of Medicine, Azhar University, Giza, Egypt; Clinical Hospital 122, Saint Petersburg, Russia; Department of Cardiology, Baqai Institute of Cardiovascular Diseases, Karachi, Pakistan; Dallah Hospital, Riyadh, Saudi Arabia; Global NIS Department, Bayer Vital GmbH, Leverkusen, Germany; Global Medical Affairs Primary Care, Bayer Pharma AG, Muellerstr. 178, S102, 6.OG, R.244, 13353 Berlin, Germany

**Keywords:** Blood pressure lowering, Combination therapy, Hypertension, Nifedipine GITS

## Abstract

**Background:**

Observational studies can provide important information on the efficacy and safety of antihypertensive agents in the real-life clinical setting. AdADOSE was a large observational study to assess the effectiveness of nifedipine GITS in combination with other antihypertensive agent(s). The study was also the first to examine the role of combination therapy with nifedipine GITS in the Middle East, Pakistan and Russia, regions that are associated with particularly high cardiovascular risk.

**Methods:**

AdADOSE was a 12-week, international, multicenter, prospective, observational study. Patients with hypertension (ie, blood pressure [BP] >140/90 mm Hg, or >130/80 mm Hg in patients at high or very high cardiovascular risk) received once-daily nifedipine GITS (30, 60 or 90 mg) in combination with another antihypertensive or as add-on to existing therapy. The primary study endpoint was the proportion of patients who achieved the target BP of <140/90 mm Hg (or <130/80 mm Hg for those at high or very high cardiovascular risk). Study outcomes are reported by descriptive statistics.

**Results:**

The study enrolled 4497 patients (*n* = 4477, safety population; *n* = 3430, efficacy population). Baseline mean systolic/diastolic BP (SBP/DBP) was 166.4/99.7 mm Hg; 85.2 % of patients had received prior antihypertensive treatment, and 90.6 % had ≥1 concomitant diseases. Following combination treatment with nifedipine GITS, target BP was achieved by 64.8 % of patients without concomitant diseases, and by 56.5 %, 32.3 % and 22.6 % with 1, 2–3 and >3 concomitant diseases, respectively. The proportion of patients achieving target BP was 51.5 % in previously untreated and 33.7 % in previously treated patients. Nifedipine GITS combination treatment provided mean SBP/DBP changes of −36.1/−18.8 mm Hg in all patients, −40.2/−21.5 mm Hg in previously untreated patients, and −35.6/−18.4 mm Hg in previously treated patients, with similar BP reductions irrespective of the number of concomitant diseases. Drug-related adverse events (AEs) were reported in 2.6 % patients. There were no serious AEs and only 0.8 % of patients discontinued due to drug-related AEs.

**Conclusions:**

Combination therapy with nifedipine GITS in a real-life observational setting was highly effective in reducing SBP/DBP in a range of hypertensive patients, with low rates of treatment-related AEs.

**Trial registration:**

Trial registration: at ClinicalTrials.gov registration number NCT01118286.

## Background

Hypertension is an established risk factor in the development of cardiovascular (CV) disease [1]. According to the World Health Organization, the worldwide prevalence of hypertension is increasing and is estimated to cause approximately 7 million premature deaths a year [2]. Clinical evidence supports blood pressure (BP) lowering to achieve defined BP targets in order to reduce the risk of CV outcomes [3, 4]. Successful attainment of adequate BP control through the selection of appropriate treatment is, therefore, of great concern to physicians and patients alike.

Several clinical trials have established the efficacy of the calcium channel blocker (CCB) nifedipine gastrointestinal therapeutic system (GITS), a dihydropyridine CCB, in the treatment of hypertension [5–8]. The Intervention as a Goal in Hypertension Treatment (INSIGHT) study showed that once-daily dosing with nifedipine GITS effectuated smooth and continuous BP control compared with conventional first-line antihypertensive therapy in the form of a diuretic [7]. A post hoc analysis of the INSIGHT data using the Framingham risk equation showed that nifedipine GITS improved CV outcomes and reduced the risk of CV events by an estimated 50 % [9].

The European Society of Hypertension (ESH)/European Society of Cardiology (ESC) guidelines recognize that most patients with elevated BP will require a combination of 2 or more antihypertensive drugs in order to achieve their BP target [10]. The combination of a CCB such as nifedipine with beta-blockers, diuretics, angiotensin-converting enzyme (ACE) inhibitors or angiotensin II receptor blockers (ARBs) has proven to be beneficial [11, 12], and guidelines recommend these CCB combinations as first-line treatment [10, 13, 14]. However, in routine clinical practice, the choice of antihypertensive agent(s) is likely to be based on the treating physician’s clinical experience and preferences. Observational studies can provide important information on the efficacy and safety of antihypertensive agents in the real-life clinical setting that is likely to reflect and inform daily practice.

This large-scale observational study (AdADOSE) assessed the effectiveness and safety of nifedipine GITS (30, 60 or 90 mg) in combination with other antihypertensive agent(s) in a real-life setting. This is also one of the first large-scale studies to investigate the benefits of nifedipine GITS in antihypertensive combination therapy in regions with high CV risk burden, including the Middle East, a high CV risk region because it has one of the highest occurrences of diabetes globally [15, 16], and Russia and Pakistan, where mortality due to CV disease is significantly higher than in Western Europe [16–18]. The study was performed in a large cohort of previously treated and untreated hypertensive patients, with and without additional CV comorbidities, reflecting the range of patients typically encountered by physicians.

## Methods

### Design

This was a 12-week, international, multicenter, prospective, observational phase IV study to monitor the effectiveness and safety of antihypertensive therapy comprising once-daily nifedipine GITS at doses of 30, 60 or 90 mg in combination with any other antihypertensive agent(s) in hypertensive patients (ClinicalTrials.gov registration number NCT01118286). Patients were enrolled from 318 clinical practices in 10 countries (Bahrain, Egypt, Jordan, Lebanon, Oman, Pakistan, Qatar, Russia, Saudi Arabia and United Arab Emirates) between January 2010 and September 2011.

Patients attended an initial visit and up to 3 follow-up clinic visits during the 12-week observation period. The timing of visits was not prespecified, but was in accordance with the treating physician’s normal practice. The decision to prescribe once-daily 30- or 60-mg nifedipine GITS tablets and to up-titrate to a dose of 90 mg or to down-titrate from a higher dose to 30 mg was made by the treating physician. The study was conducted using approved country-specific prescribing information for the study drugs. It was also performed in accordance with the guidelines of the European Medicines Agency and the US Food and Drug Administration, and was conducted in accordance with the Declaration of Helsinki (1996) and the ICH Harmonised Tripartite Guideline for Good Clinical Practice (GCP). Ethics committee approval for this study was provided in Pakistan by the Research Ethics Committee of the Ministry of Health, and in Russia by the Ethics Committee of the Federal Service on Surveillance in Healthcare and Social Development. No ethics committee approval was required in Bahrain, Egypt, Jordan, Lebanon, Oman, Qatar, Saudi Arabia and the United Arab Emirates at the time of study conduct.

Written informed consent (in accordance with GCP and local legislation) was provided by all patients prior to any study-specific investigations.

### Patients

Male or female patients aged ≥18 years with essential hypertension (ie, BP >140/90 mm Hg or >130/80 mm Hg in patients at high or very high CV risk) [10] were eligible for enrollment. Patients could be either previously untreated or insufficiently controlled on current antihypertensive regimens not containing CCBs. Patients with contraindications to nifedipine GITS (as stated in the local product information) were not considered suitable for nifedipine GITS treatment by the treating physician and were, therefore, excluded.

### Observational parameters

This study had the primary aim to assess the proportion of patients reaching the target BP of <140/90 mm Hg (or <130/80 mm Hg for those at high or very high CV risk), in accordance with ESH/ESC guidelines current at the time of study [10]. Systolic BP (SBP), diastolic BP (DBP), pulse pressure and heart rate were measured at the initial study visit and at the follow-up visits using standard cuff BP measuring equipment, according to local practice. A single BP measurement was the standard approach in the participating countries, with the exception of Egypt, where a mean of 3 measurements taken a few minutes apart was used. Achievement of target BP in this manuscript is a composite of the proportion of patients not at high/very high CV risk achieving their BP target of <140/90 mm Hg and the proportion of patients at high/very high CV risk achieving their BP target of <130/80 mm Hg.

All adverse events (AEs) that occurred throughout the study period were recorded. AEs were classified using the *Medical Dictionary for Regulatory Activities* (*MedDRA*) version 14.1, and were evaluated for seriousness, relation to study drug, action taken and outcomes.

Efficacy and tolerability were also evaluated in the physician’s final assessment of therapy. All data were recorded in standardized case report forms.

### Statistical analyses

Descriptive summary statistics for categorical and quantitative (continuous) data were reported. Percentage values were calculated as the proportion of each category including the category of missing values. Analyses were also stratified according to age, sex, pretreatment status, initial BP and concomitant diseases and these data were used for the CV risk stratification of the patients. Primary safety analyses were performed on the full analysis set, which consisted of all enrolled patients who took any dose of study medication in combination with any other antihypertensive agent(s) (except CCBs) who had an initial BP measurement and at least 1 follow-up BP measurement. The efficacy evaluation was performed on all patients with at least 1 follow-up BP measurement.

## Results

### Patient disposition and baseline characteristics

**Fig. 1:**
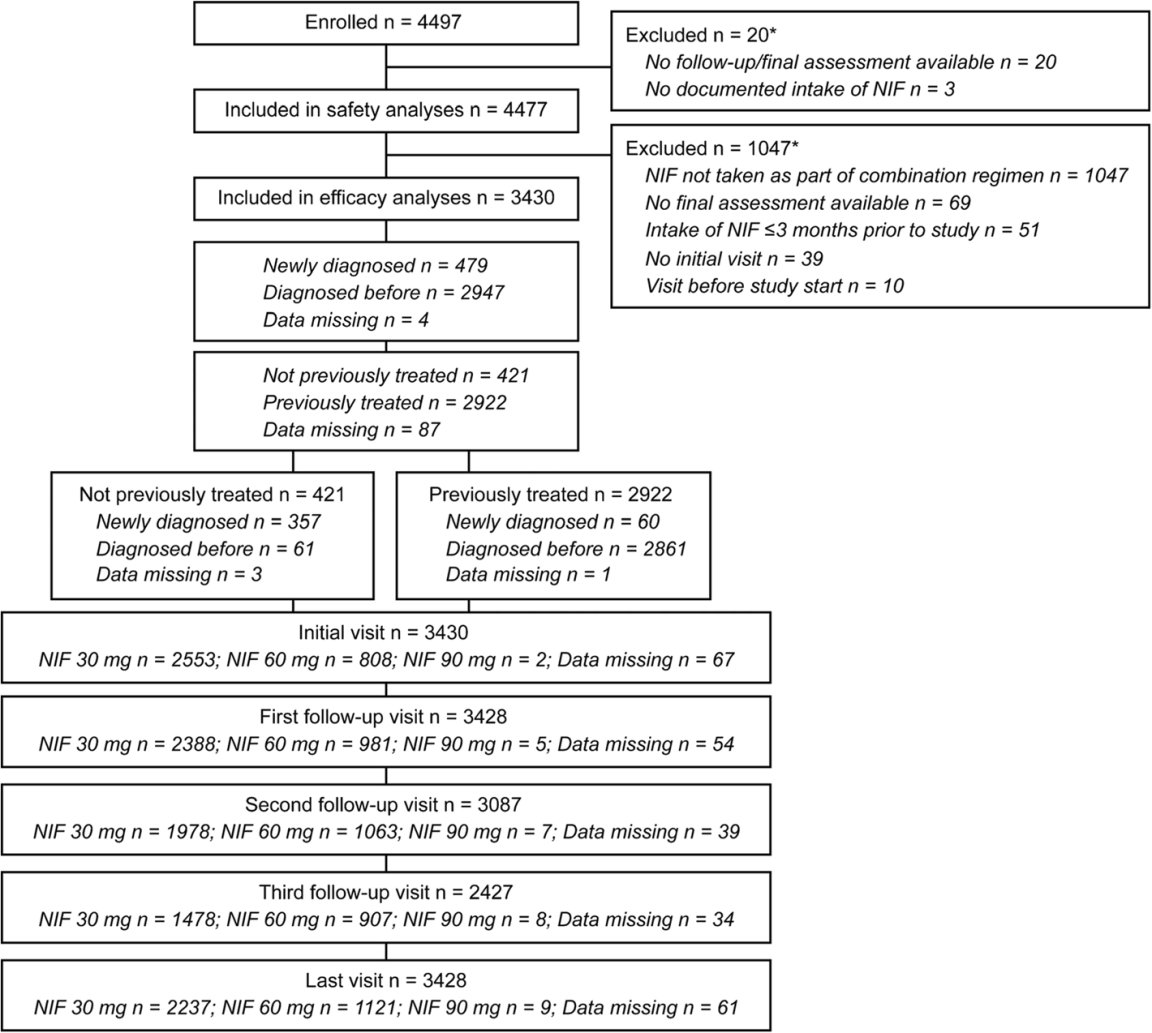
Study design. Disposition of patients treated with nifedipine GITS once daily as part of combination antihypertensive treatment regimens. GITS = gastrointestinal therapeutic system; NIF = nifedipine GITS. Note: * Information on exclusions overlaps

A total of 4497 patients were enrolled in the study, and 4477 (99.6 %) were included in the safety analysis. Of these 4477 patients, 1047 (23.4 %) patients were excluded since they had received nifedipine GITS as a monotherapy only, leaving 3430 (76.6 %) evaluable for the efficacy analysis. The reasons for study exclusions are summarized in Fig. 1. Baseline characteristics for the efficacy population are presented in Table 1. Patients were enrolled from Egypt (*n* = 939), Pakistan (*n* = 749), Saudi Arabia (*n* = 650), Russia (*n* = 461), Lebanon (*n* = 229), United Arab Emirates (*n* = 127), Qatar (*n* = 96), Jordan (*n* = 85), Bahrain (*n* = 61), and Oman (*n* = 33).

**Table 1:** Patient baseline characteristics, efficacy population

Characteristic	n (%)
Patients, n (%)	3430 (100)
Gender, n (%)	
Male	1993 (58.1)
Female	1387 (40.4)
Mean age, years ± SD (range); *n* = 3322	53.4 ± 10.4 (19.0–89.0)
Age, n (%)	
<65 years	2836 (82.7)
≥65 years	486 (14.2)
Race, n (%)	
Asian	1212 (35.3)
White	966 (28.2)
Black	96 (2.8)
Other	970 (28.3)
Mean BMI, kg/m^2^ ± SD (range); *n* = 3214	29.9 ± 5.2 (15.1–57.4)
Mean BP, mm Hg ± SD (range)	
SBP [*n* = 3413]	166.4 ± 16.7 (100.0–260.0)
DBP [*n* = 3418]	99.7 ± 9.9 (55.0–170.0)
Stages of hypertension, n (%)^a^	
Normal	8 (0.2)
High normal	16 (0.5)
Grade 1 (mild)	407 (11.9)
Grade 2 (moderate)	1620 (47.2)
Grade 3 (severe)	1142 (33.3)
Isolated systolic hypertension	218 (6.4)
Duration of hypertension, n (%); *n* = 3426	
Newly diagnosed	479 (14.0)
Previously diagnosed	2947 (85.9)
<1 year	434 (14.7)
1–5 years	1210 (41.1)
6–10 years	692 (23.5)
>10 years	554 (18.8)
Previous antihypertensive therapy, n (%)	
0	421 (12.3)
1	1314 (38.3)
2	1067 (31.1)
≥3	521 (15.2)
Previous classes of antihypertensive therapy, n (%); *n* = 2922^b^	
ACE inhibitors	1210 (41.4)
ARBs	833 (28.5)
CCBs^c^	348 (11.9)
β-blockers	1389 (47.5)
Thiazide diuretics	940 (32.2)
Other	97 (3.3)
Smoking history, n (%)	
Current smoker	725 (21.1)
Never	1940 (56.6)
Past smoker	606 (17.7)

Data for all variables were not available for all patients

*ACE* angiotensin-converting enzyme, *ARBs* angiotensin II receptor blockers, *BMI* body mass index, *CCBs* calcium channel blockers, *DBP* diastolic blood pressure, *GITS* gastrointestinal therapeutic system, *SBP* systolic blood pressure, *SD* standard deviation

^a^Definitions according to ESH/ESC 2007 guidelines. ^b^Multiple responses possible. ^c^Other than nifedipine GITS

At the initial visit, 2097/3430 (61.1 %) patients were classified as being at high or very high CV risk [10]. General concomitant diseases/conditions were reported in 92.3 % of the patients and specific CV concomitant diseases/conditions in 90.6 % patients, including dyslipidemia (41.3 %), obesity (26.7 %) and fatty liver (26.7 %) (Table 2). A total of 2922/3430 (85.2 %) patients had received previous antihypertensive treatment.

**Table 2:** Specific concomitant diseases at baseline

	Patients (*n* = 3430)
Patients with specific concomitant diseases, n (%)	3109 (90.6)
Dyslipidemia	1417 (41.3)
Obesity	917 (26.7)
Fatty liver	917 (26.7)
Stable angina pectoris	445 (13.0)
Diabetic neuropathy	396 (11.5)
Microalbuminuria	275 (8.0)
Diabetic retinopathy	271 (7.9)
Myocardial infarction	230 (6.7)
Renal insufficiency	167 (4.9)
Peripheral vascular disease	158 (4.6)
Congestive heart failure	141 (4.1)
Coronary revascularization	137 (4.0)
Transient ischemic attack	115 (3.4)
Cerebrovascular accident	97 (2.8)
Number of concomitant diseases, n (%)^a^	
1	622 (18.1)
2	863 (25.2)
3	685 (20.0)
4	442 (12.9)
5	264 (7.7)
6	149 (4.3)
7	82 (2.4)
8	34 (1.0)
9	12 (0.3)
10	5 (0.1)
>10	8 (0.2)

^a^Coded according to Medical Dictionary for Regulatory Activities System Organ Class

2427/3430 (70.8 %) patients attended 4 clinic visits, 660 (19.2 %) attended 3 and 341 (9.9 %) attended 2 visits during the study. For 2 (0.1 %) patients, the date(s) for the follow-up visit(s) were missing. However, these 2 patients were included in the efficacy analyses, as there was a final assessment at the end of the observation period. The average time between visits was approximately 4 weeks.

### Treatment

The majority of patients (2073/3430 [60.4 %]) received a constant daily dose of 30-mg nifedipine GITS during the study period; another 686 (20.0 %) patients received a constant daily dose of 60-mg nifedipine GITS, and 392 (11.4 %) had their daily dose adjusted from 30-mg to 60-mg nifedipine GITS during the study period. Doses were down-titrated to 30-mg nifedipine GITS in 97 patients (2.9 %). Nine (0.3 %) patients completed the study taking 90-mg nifedipine GITS.

Details on the concomitant drugs prescribed by physicians were also recorded at visits. At the initial visit, most patients were taking 1 (1792 [52.2 %]) or 2 (1053 [30.7 %]) concomitant antihypertensive drugs, while 369 (10.8 %) and 55 (1.6 %), respectively, took 3 or 4 concomitant antihypertensive drugs, and 9 patients (<0.3 %) took 5 or 6 concomitant antihypertensive drugs. 152 patients (4.4 %) took no concomitant antihypertensive drug at the study start but did so during the observation period, and were therefore included in the analysis. At the last visit, the proportion of patients taking 1 concomitant antihypertensive drug had fallen to 49.8 % (1707) whereas the proportion taking no additional antihypertensive drug increased to 7.0 % (240). The latter patients were also included in the efficacy analysis since they were prescribed antihypertensive combination therapy at some point during the study period.

Selection of other antihypertensive medications (and doses) was also at the discretion of the treating physician, and reflected daily practice. While assessment of comedication use was not a primary objective of the study, the most frequently reported concomitant antihypertensive drugs at both initial and final visits were beta-blockers (43.6 % and 43.7 % of patients, respectively), followed by ACE inhibitors (32.2 % and 31.5 %) and thiazide diuretics (31.0 % and 30.3 %). The most frequent non-antihypertensive concomitant medications were acetylsalicylic acid (28.3 % of patients), atorvastatin calcium (13.8 %) and metformin (11.4 %).

### Efficacy

Overall, the target BP goal was reached by 36.3 % (1246/3428) of patients treated with nifedipine GITS combination therapy. The proportion of patients not at high/very high CV risk who achieved the target BP of <140/90 mm Hg was 70.8 % (942/1331), whereas the proportion of patients at high or very high risk who achieved the lower BP target of 130/80 mm Hg was only 14.5 %. When patients who met the target BP (ie, <140/90 mm Hg, or <130/80 mm Hg in those at high or very high CV risk) were stratified according to specific CV concomitant diseases, the respective target BP was achieved by 64.8 % in patients with no concomitant disease, and by 56.5 %, 32.3 % and 22.6 % with 1, 2 or 3 and >3 concomitant diseases, respectively (Table 3). The BP target was achieved in 51.5 % of patients who were previously untreated and in 33.7 % of those who had previously received antihypertensive medication (Table 3).

**Table 3:** Blood pressure goal

	Patients reaching BP goal, n (%)	
	1st follow-up visit	Last visit
Overall population; *n* = 3428^a^	542 (15.8)	1246 (36.3)
Previous antihypertensive medication		
No; *n* = 421	86 (20.4)	217 (51.5)
Yes; *n* = 2920	431 (14.8)	985 (33.7)
Concomitant disease		
0; *n* = 264	82 (31.1)	171 (64.8)
1; *n* = 621	177 (28.5)	351 (56.5)
2–3; *n* = 1547	213 (13.8)	499 (32.3)
>3; *n* = 996	70 (7.0)	225 (22.6)

Proportion of patients reaching target BP following treatment with nifedipine GITS in combination with other antihypertensive agents

*BP* blood pressure, *GITS* gastrointestinal therapeutic system

^a^BP <140/90 mm Hg (or BP <130/80 mm Hg in those at high or very high CV risk)

**Fig. 2:**
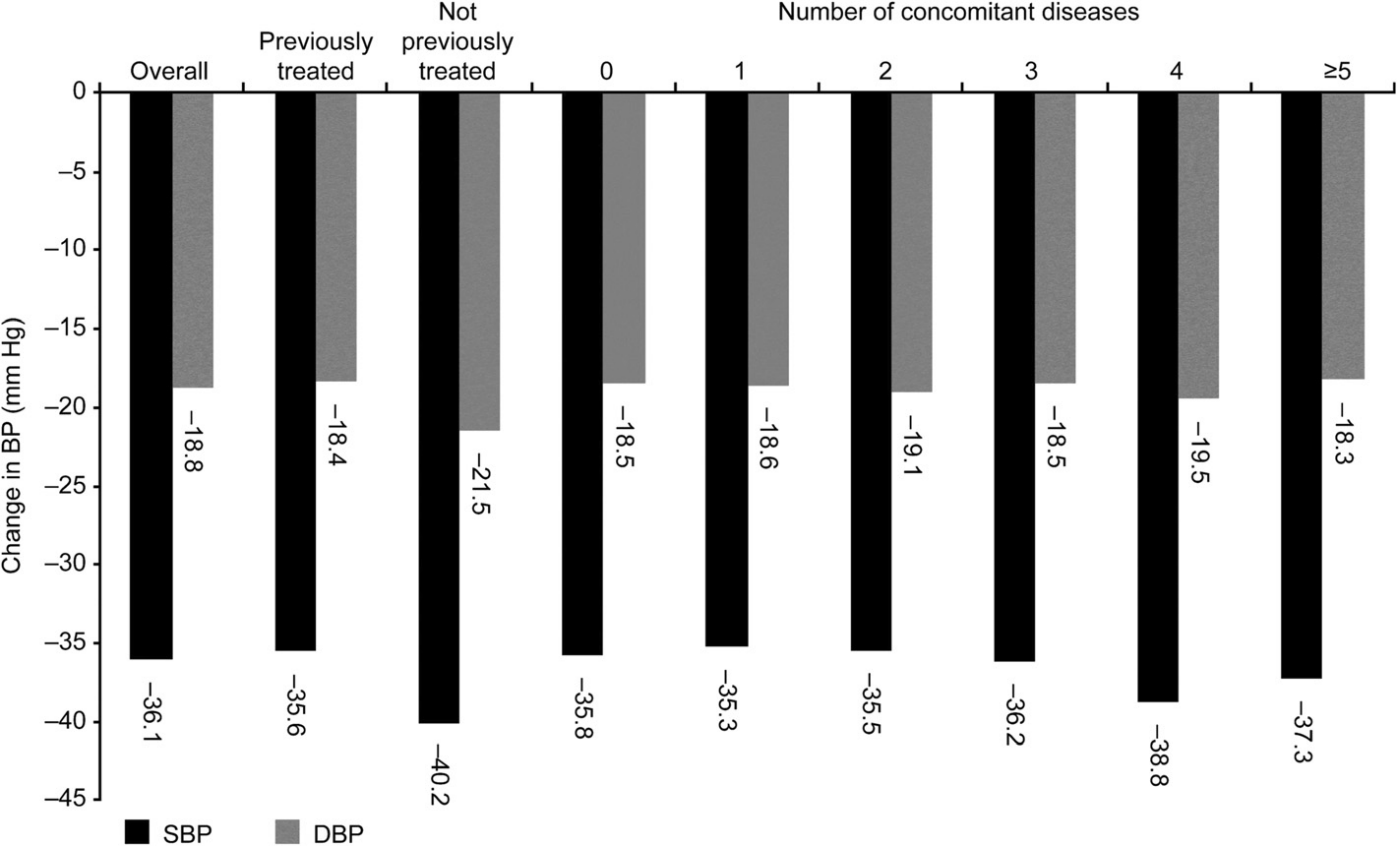
Change in SBP and DBP. Mean absolute difference in SBP and DBP in patients who had or had not previously received antihypertensive medication and according to number of concomitant diseases (initial to last visit) following 12 weeks’ treatment with nifedipine GITS once daily as part of combination antihypertensive regimens. DBP = diastolic blood pressure; GITS = gastrointestinal therapeutic system; SBP = systolic blood pressure

Combination therapy with nifedipine GITS provided a mean absolute reduction in SBP/DBP of −36.1/–18.8 mm Hg (Fig. 2). BP reductions were similar irrespective of the number of concomitant diseases present, with mean SBP reductions ranging from 35.3 to 38.8 mm Hg, and mean DBP reductions ranging from 18.3 to 19.5 mm Hg during the study (Fig. 2). Mean absolute reductions were −40.2/−21.5 mm Hg in previously untreated and −35.6/−18.4 mm Hg in previously treated patients.

**Fig. 3:**
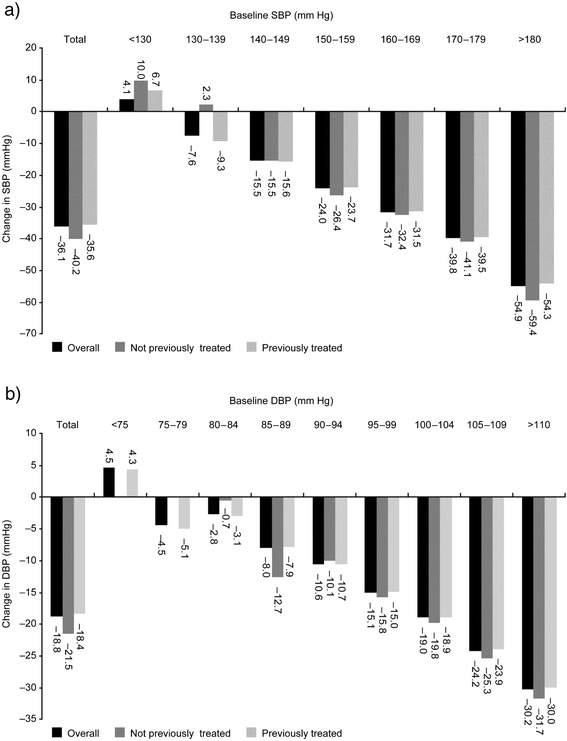
Change in SBP and DBP. Mean absolute difference in SBP (**a**) and DBP (**b**) according to baseline SBP and DBP following 12 weeks’ treatment (initial to last visit) with nifedipine GITS once daily as part of combination antihypertensive regimens. DBP = diastolic blood pressure; GITS = gastrointestinal therapeutic system; SBP = systolic blood pressure

When patients were stratified according to initial BP values, mean BP reductions were greater for higher baseline SBP (Fig. 3a) and DBP (Fig. 3b). In addition, time course analyses showed that rates of systolic hypertension (ie, SBP ≥140 mm Hg) decreased steadily during study from 97.8 % at initial visit to 22.8 % by the last visit, as did diastolic hypertension (ie, DBP ≥90 mm Hg; from 92.6 to 13.0 %). The decreases in rates of systolic and diastolic hypertension during the study occurred in both previously treated and untreated patients (Table 4). Isolated systolic hypertension (ie, ≥140/<90 mm Hg) was recorded in 6.4 % (218) of patients at the initial visit; this increased to 20.0 % (686) by the first follow-up visit and then decreased to 14.4 % (493) by the last visit.

**Table 4:** Systolic and diastolic hypertension

	SBP <140 mm Hg		DBP <90 mm Hg	
	Initial visit	Last visit	Initial visit	Last visit
Overall population, n (%); *n* = 3430	60 (1.7)	2644 (77.1)	242 (7.1)	2975 (86.8)
No previous antihypertensive medication, n (%); *n* = 421	4 (1.0)	343 (81.5)	10 (2.4)	368 (87.4)
Previously received antihypertensive medication, n (%); *n* = 2922	50 (1.7)	2238 (76.6)	220 (7.5)	2539 (87.0)

Proportion of patients with SBP <140 mm Hg or DBP <90 mm Hg following treatment with nifedipine GITS in combination with other antihypertensive agents

*DBP* diastolic blood pressure, *GITS* gastrointestinal therapeutic system, *SBP* systolic blood pressure

Between the initial and last visit, the mean pulse pressure (± SD) decreased from 66.7 ± 14.9 mm Hg to 49.3 ± 9.3 mm Hg, and the mean heart rate (± SD) decreased from 78.6 ± 10.8 bpm to 75.5 ± 7.7 bpm. Changes in these parameters were similar for patients regardless of the use of previous antihypertensive medication. In patients who had not previously received antihypertensive medication, mean pulse pressure decreased from 67.3 ± 15.1 mm Hg to 48.4 ± 8.7 mm Hg, and mean heart rate decreased from 81.8 ± 9.5 bpm to 76.4 ± 7.5 bpm. In patients previously treated with antihypertensive medication, mean pulse pressure decreased from 66.6 ± 14.8 mm Hg to 49.4 ± 9.4 mm Hg, and mean heart rate decreased slightly from 78.0 ± 10.9 bpm to 75.4 ± 7.8 bpm.

### Tolerability

A total of 142/4477 (3.17 %) patients experienced 201 AEs during the study period. The most common AEs were peripheral edema (54 patients [1.2 %]) and headache (28 [0.6 %]) (Table 5). Overall, 166 events in 114/4477 (2.6 %) patients were considered by the treating physician to be study-drug related, including peripheral edema (48 patients [1.1 %]) and headache (26 [0.6 %]). No serious AEs or serious study-drug–related AEs were reported. Forty-eight AEs in 35 (0.8 %) patients resulted in permanent discontinuation of study drug; peripheral edema (14 [0.3 %]) was the most common AE leading to treatment discontinuation.

**Table 5:** AEs and drug-related AEs

	AEs	Drug-related AEs
Total, patients (%); *n* = 4477	142 (3.2)	114 (2.6)
Peripheral edema, n (%)	54 (1.2)	48 (1.1)
Headache, n (%)	28 (0.6)	26 (0.6)
Edema, n (%)	15 (0.3)	9 (0.2)
Palpitations, n (%)	11 (0.3)	9 (0.2)
Flushing, n (%)	10 (0.2)	10 (0.2)
Constipation, n (%)	5 (0.1)	5 (0.1)
Dizziness, n (%)	5 (0.1)	5 (0.1)

Incidence of AEs and drug-related AEs (≥0.1 % incidence) in patients during treatment with nifedipine GITS in combination with other antihypertensive agents

*AEs* adverse events, *GITS* gastrointestinal therapeutic system

### Physician satisfaction with nifedipine GITS combination treatment

Treating physicians assessed the efficacy of nifedipine GITS combination treatment as “very good” in 64.2 % (2203/3430) of patients, “good” in 30.2 % (1036), “sufficient” in 3.9 % (133) and “insufficient” in 1.1 % (38), with missing data in the remainder. The tolerability of nifedipine GITS combination treatment was assessed as “very good” in 58.0 % (1991), “good” in 35.7 % (1224), “sufficient” in 3.3 % (112) and “insufficient” in 0.8 % (29), with missing data in the remainder. Overall, treating physicians were “very satisfied” in 57.7 % (1978) and “satisfied” in 38.2 % (1311) of patients with the therapeutic effect of nifedipine GITS combination treatment; 1.7 % (58) were “unsatisfied” and 0.1 % (3) were “very unsatisfied,” with missing data in the remainder. Physicians intended to continue with nifedipine GITS combination treatment beyond the study period in 93.4 % (3202) of patients.

## Discussion

The aim of this observational study was to monitor the effectiveness and safety of nifedipine GITS combination therapy when used in routine clinical practice in countries in the Middle East, Pakistan and Russia. In this large cohort of hypertensive patients, the majority (85 %) had previously received antihypertensive treatment, usually with 1 or 2 antihypertensive therapies; beta-blockers (48 %) and ACE inhibitors (41 %) were the most common medications. Despite the high level of pretreatment, nearly one half of the patients (47 %) had SBP 160–179 mm Hg and one-third (33 %) had SBP ≥180 mm Hg at study baseline. Many patients had additional CV risk factors, leading to over one half (61 %) of patients being classified as at high CV risk. During the study period, the combination of nifedipine GITS with other antihypertensive medications resulted in a mean BP reduction of −36.1/−18.8 mm Hg in all patients. This clinically significant reduction was consistent in patients stratified according to baseline antihypertensive medication and irrespective of the number of concomitant diseases. Greater BP reductions were observed in patients with higher baseline BP.

A recent large-scale observational study across the Far East, the Middle East, Pakistan and Mexico, with nifedipine GITS 30 mg or 60 mg, given either as monotherapy (51.8 % of patients) or as add-on (48.2 % of patients) to antihypertensive therapy, reported an overall mean BP reduction of −28/−14 mm Hg. The extent of BP reduction was associated with hypertension grade, age, presence of ≥5 additional CV risk factors, as well as prior treatment [19]. Overall, target BP (<140/90 mm Hg; 130/80 mm Hg in patients with diabetes) was achieved in 29.5 % of patients. The BP target achievement rate was slightly higher in those receiving nifedipine GITS as monotherapy (33.4 %) than in those receiving nifedipine GITS as part of an antihypertensive combination regimen (25.3 %), which may have been because the patients requiring combination therapy had more severe degrees of hypertension. Other, smaller observational studies in China have similarly shown the BP-lowering efficacy of nifedipine GITS [20, 21].

The findings from the present observational study confirm that nifedipine GITS in initial combination therapy or as add-on therapy improves the achievement of BP treatment goals (based on ESH/ESC guidelines at the time of study) in a broad spectrum of patients. In those without additional comorbidities, an additional 34 % of patients reached target BP (from 31 to 65 %) after 12 weeks of nifedipine GITS treatment. Nifedipine GITS combination therapy was also effective in patients with numerous (≥3) concomitant diseases, where the BP target achievement rate was improved by 16 % (from 7 to 23 %). The reduction in SBP and DBP observed in our study is reflected by the substantial shifts from higher to lower hypertension grades. The proportion of patients with SBP <140 mm Hg or DBP <90 mm Hg increased substantially during the study (from <2 % and 7 % at the initial visit to 77 % and 87 %, respectively). Again, these observations were consistent in both previously treated and untreated patients.

A limitation of the AdADOSE study is that a stricter treatment target was used for patients at high or very high CV risk (<130/80 mm Hg) compared with the goal in other patients (<140/90 mm Hg). The lower target is notoriously difficult to achieve in clinical practice and may not even be beneficial, as suggested by the 2009 reappraisal of the ESH/ESC guidelines [13]. The ESH/ESC guidelines were updated in 2013, and now recommend a target of <140/90 mm Hg for all patients with an optimal DBP of between 80 and 85 mm Hg for diabetic patients [22]. The UK NICE guidelines also recommend a BP target of <140/90 mm Hg for all hypertensive patients [14]. If this BP target of <140/90 mm Hg were applied to all patients in this study, irrespective of CV risk status, the proportion of patients achieving BP control would increase by 40 percentage points—to 72 %—with nifedipine GITS combination therapy. This high rate of achievement of the newer single BP target of 140/90 mm Hg was also not affected by the presence of concomitant diseases, since 69.7 % of all patients without concomitant disease would have achieved this target BP, as well as 69.7 %, 71.6 % and 76.0 % of patients with 1, 2–3 or >3 concomitant diseases, respectively [23]. These data demonstrate the efficacy of a nifedipine GITS-containing combination therapy and compare favorably with data from other studies such as the National Health and Nutrition Examination Survey (NHANES), where only 31.0 % of the overall treated population of hypertensive patients achieved control defined as <140/90 mm Hg [24].

A further limitation of this study is the classification of patients at high or very high CV risk. On the case report forms used, patients with diabetes were reported as such only if they had diabetic retinopathy or diabetic neuropathy, so it is likely that more patients had diabetes in this study than were reported. Based on this, it is difficult to classify patients with confidence according to their CV risk, and a greater emphasis has therefore been placed in the interpretation of outcomes on the number of concomitant diseases. Other limitations pertain to the nature of the study design; being observational, there was no control group, and the specific contributory effects of factors such as the types (and doses) of concomitant medications used, the selection of nifedipine GITS dose (30 mg or 60 mg), and the patient ethnicity is unknown. It should be noted that a detailed assessment of the impact of concomitant medications was not an objective of this observational study. Prescribing low-dose combination therapy, rather than up-titration of monotherapy, is in accordance with guideline recommendations; however, the dose of the medication prior to change was not recorded, and it is not possible to determine if it was maximal. Additionally, the effect of potential suboptimal treatment regimens is unknown, especially in high CV risk patients. Finally, the potential influence of “white-coat hypertension” on reported outcomes cannot be determined in this study [25].

The profile of AEs in AdADOSE (eg, edema and headache) was in agreement with the known safety profile of nifedipine GITS. The incidence of drug-related AEs (2.6 % of patients) was lower than expected from reference safety and tolerability information [5, 7, 19–21, 26]. This may suggest a reporting bias in the current study, with investigators tending to underreport mild to moderate AEs that are known to be associated with nifedipine GITS therapy. Underreporting of mild/moderate AEs is a recognized feature of observational studies. In terms of treatment satisfaction, physicians rated the overall efficacy and tolerability of nifedipine GITS as very good or good in 94 % of patients, and they rated their satisfaction with therapeutic efficacy as very good or good for 96 %. For just over 93 % of patients in this study, the physicians intended to continue antihypertensive combination therapy containing nifedipine GITS.

The large observational AdADOSE study performed in patients from countries with high CV risk burden provides results that are relevant to real-world practice. Nifedipine GITS 30 mg or 60 mg once daily in combination therapy is highly effective in the treatment of hypertension, irrespective of the presence of concomitant disease, and is generally well tolerated with a very low incidence of AEs. This study therefore confirms the efficacy and safety profile of nifedipine GITS that was demonstrated in earlier clinical trials and supports its value in the treatment of patients with hypertension including those with additional CV risk factors and comorbidities. The study also highlights the importance of combination therapy in a real-life setting to improve BP control rates.

## Conclusion

Combination therapy with nifedipine GITS in a real-life observational setting was highly effective in reducing SBP/DBP in a range of hypertensive patients, with low rates of treatment-related adverse events.

## Abbreviations

ACE: Angiotensin-converting enzyme
AE: Adverse event
ARBs: Angiotensin II receptor blockers
BP: Blood pressure
CCB: Calcium channel blocker
CV: Cardiovascular
DBP: Diastolic blood pressure
ESC: European Society of Cardiology
ESH: European Society of Hypertension
GITS: Gastrointestinal therapeutic system
INSIGHT: Intervention as a Goal in Hypertension Treatment study
NHANES: National Health and Nutrition Examination Survey
SBP: Systolic blood pressure
SD: Standard deviation
